# Supplementary material for: Are carbon emissions trading and green financial instruments synergistic? -Comprehensive quantitative research based on content analysis
Source: PLoS One. 2024 Mar 7;19(3):e0298601. doi: 10.1371/journal.pone.0298601 (PMC10919601; doi:10.1371/journal.pone.0298601)
Supplement: S1 Data — (ZIP) [file pone.0298601.s001.zip › Data/地级市Z_F工作报告.zip!/╔┬╬≈/╬╝─╧╩╨/└┤╫╘╣1⁄2╓┌║┼í╛┬φ┐╦╩2╛▌═°í┐_2015.docx]

各位代表：

　　现在，我代表市人民政府向大会作工作报告，请予审议，并请各位政协委员和列席会议的同志提出意见。

　　一、四届人大六次会议确定任务完成情况

　　过去的一年，在市委的正确领导下，市政府团结依靠全市人民，认真贯彻落实党的十八大和十八届三中、四中全会精神，牢牢把握稳中求进、提质增效的工作总基调，积极适应发展新常态，坚持城区、园区和景区协调推进，新型工业化、信息化、城镇化和农业现代化互促共动，全市经济社会继续保持平稳较快发展态势。生产总值完成1460.9亿元，增长10.5%，高于全省0.8个百分点；地方财政收入完成67.5亿元，同口径增长15%；城乡居民收入分别达到26725元和8534元，增长10.6%和12.8%。

　　一是投资持续较快增长，发展后劲不断增强。全社会固定资产投资完成1765.6亿元，增长20.3%。280个市级重点项目完成投资1263.7亿元，国道108渭南至汉村段升等改造等33个项目建成投用，渭玉高速等76个项目超额完成年度投资任务，沿黄公路渭洛河大桥等40个前期项目提前开工建设。渭南热电联产、富平热电联产等项目相继获批，秦电8号机组通过国家核准。成功举办了第二届黄河金三角投资合作交流大会和3D打印暨医疗器械产业化推进会，全年签订招商引资合同项目335个，到位资金570亿元，增长12.9%。

　　二是产业转型步伐加快，经济质量稳步提升。富平生态水泥、蒲城70万吨烯烃等一批重大工业项目建成投产，陕西黑猫焦化成功上市，3D打印产业培育基地建设迈出实质性步伐，市中小企业服务中心被认定为国家中小企业公共服务示范平台。淘汰小水泥、小钢铁等落后产能185万吨，关闭烟花爆竹生产点104家。工业园区发展到20个，新增规模以上工业企业55家，规模以上工业总产值、增加值分别增长8.1%和12.3%。现代农业园区发展到346个，市级以上龙头企业达到357家。“大荔冬枣”、“蒲城西瓜”取得国家地理标志认证，富平荣获“中国羊乳之都”称号，白水被评为“中国有机苹果第一县”。大华山旅游目的地建设扎实推进，“史记韩城·风追司马”、少华山、潼关古城、合阳洽川、富平陶艺村等景区建设全面提升，潼关国家级黄金矿山公园通过评审，华阴荣获“中国十大生态文化旅游城市”称号，全市接待游客3200万人次，实现旅游综合收入252亿元，增长19.7%。信达商业街加快建设，万达城市综合体主体完工，湭河老街美食城一期建成运营，电子商务企业蓬勃发展，我市被确定为全国农产品现代流通网络建设试点市。新增限额以上贸易企业101家，全社会消费品零售总额完成418.6亿元，增长14%。新引进、设立金融机构5家，全市金融机构存款余额1664.6亿元，贷款余额811.4亿元，分别增长9.3%和11.8%。第三产业增加值增长9.7%，增速位居全省第一。

　　三是城乡面貌深刻变化，生态建设成效明显。中心城市首位度不断提升，实施建设项目190个，完成投资200亿元。打通了6条“断头路”，实施了三座大桥及湭河亮化，完成了民生路中段拓宽改造及连霍高速西出入口改造主体工程，渭河城区段退耕还河还湿地初见成效。县城建设全面提升，形成了大荔同州湖、富平两河一路综合改造、合阳省级文明县城创建达标等新的亮点。19个省市重点镇和文化旅游名镇实施项目130个，完成投资21.9亿元。12个市级新型农村社区完成投资3.9亿元。108个美丽乡村示范村建设基本达标，洽川等4镇5村被命名为省级生态镇村，大荔美丽乡村建设受到省上肯定。秦东大地园林化工程新增绿地74.2万亩，白水林皋湖、富平石川河被确定为国家级湿地公园试点，蒲城被评为全国生态工程示范县。渭河治污三年行动圆满收官，“治污降霾·保卫蓝天”行动扎实有效，在全省地级市中率先引进纯电动公交车24辆，市区二级以上优良天数达到220天，超出省考目标80天。二氧化硫、化学需氧量、氨氮和氮氧化物等四项减排任务超额完成，万元GDP能耗下降4.3%。

　　四是民生改善力度加大，安全稳定得到加强。全年财政民生支出242.9亿元，占财政支出的82.8%。备受群众关注的市体育中心、文化艺术中心、市民综合服务中心等重大民生工程建成投用，中心城市供热“煤改气”投入运行，新增供热面积110万平方米。抽黄供水工程试验段基本建成，多年未解决的乡镇机关供暖设施改造全面完成。新增城镇就业7.2万人，启动建设扶贫移民搬迁安置社区（村）48个、涉及1.4万人，实现脱贫18.6万人。33.5万人饮水安全得到解决，30.4万城乡低保人口应保尽保，城乡居民养老参保277万人，基本建成各类保障房5.47万套，农民进城落户13.7万人。库区移民后期扶持政策持续实施，移民生产生活条件进一步改善。渭南中学、渭南初级中学和渭南青少年体育学校建成投用，改建公办幼儿园31所，城区“大班额”问题基本解决，大学区改革受到中省肯定。合阳通过国家义务教育均衡县验收，韩城通过省级“双高双普”验收，渭南技师学院被确定为国家级高技能人才培训基地。9个县市列入国家县级公立医院综合改革试点，澄城医改得到国家肯定，城乡居民医疗保险政府补助标准提高到每人每年350元，单独两孩政策平稳实施。国家公共文化服务体系示范区创建深入推进，群众文体活动丰富多彩，市第十二届运动会成功举办，《天国的百合花》荣获全国秦腔艺术节优秀剧目奖。地方志第二轮修志工作顺利完成，智慧城市建设稳步推进，我市列入全国公交一卡通试点城市，潼关在全省率先实施农村免费数字电视。“六五”普法通过中省中期验收，信访“三无”创建得到加强，临渭区医患调解经验在全国推广。安全生产力度进一步加大，平安渭南建设扎实推进，公众安全感持续上升，社会大局和谐稳定。

　　五是改革创新卓有成效，对外开放不断扩大。积极推进简政放权改革和“权力清单”制度，分三轮先后下放、取消、转移市级经济社会管理权限1171项，市场活力充分释放。食品药品监管体制改革成果进一步巩固，工商登记制度改革成效明显。市县行政机构改革方案报省上审批，镇村综合改革全面铺开，县市区事业单位清理规范工作取得阶段性成果。国企国资改革稳步推进，市属经营性国有资产基本实现集中统一监管。工业企业改制遗留问题化解和商业企业改制基本完成，公务用车改革在全省率先启动，惠民补贴“一卡通”担保贷款惠及群众5万多户，农村土地确权等全面推进。市科技资源统筹中心建成运行，韩城被确定为全省第一批创新型县市试点，“大荔模式”实现乡镇全覆盖。全市科技研发投入占GDP比重达到1.31%，技术合同交易额突破1亿元，招引高层次人才346名。渭南海关和出入境检验检疫局正式开关开检，我市与韩国龟尾市缔结为友好城市，西渭融合发展、黄河金三角区域合作等进一步加强。

　　六是政府自身建设全面提升，干部作风切实改进。党的群众路线教育实践活动和纪律作风大整顿大排查、“当好标杆，向我看齐”等活动深入开展，全市集中整改“四风”问题3974个，会议经费、“三公”经费支出分别下降43.5%和21.7%。机关效能建设向纵深拓展，“百姓问政”、“向人民报告”、邀请公民进政府等活动受到群众好评。依法行政示范市通过省级验收，人民网留言办理被评为全国示范市。行政监察、审计监督等工作力度持续加大。自觉接受市人大、市政协监督和社会监督，积极回应人大代表、政协委员对社会热点问题的关注，市人大代表建议和政协提案全部按期办结答复，办理质量明显提高。

　　同时，国防动员、双拥工作、国家安全、人民防空、民族宗教、外事侨务不断加强，妇女、儿童、老龄、残疾人、红十字事业全面进步，统计、气象、地震、档案、新闻出版和广播电视等各项工作都取得了新的成绩。

　　各位代表，过去的一年，在经济下行压力加大、面临诸多不利因素的情况下，全市经济社会发展稳中有进、稳中趋好，主要指标增速均高于全省平均水平，经济总量首次进入全省前五；渭南3D打印产业化培育基地破土而生，华山旅游门票收入首次突破3亿元；简政放权、医改、教育等多项工作受到国家表彰，公共文化服务体系示范区创建、食品药品监管体制改革、工商登记制度改革、公务用车改革等走在了全省前列。这些成绩的取得，是市委正确领导的结果，是市人大、市政协和社会各界监督支持的结果，是全市人民团结奋斗的结果。在此，我谨代表市人民政府，向全市广大干部群众、驻渭部队指战员、武警官兵和公安干警致以崇高的敬意！向大力支持政府工作的各位代表、各位委员和各界人士表示衷心的感谢！

　　在看到发展成绩的同时，我们也清醒地看到，受经济下行以及市场因素等的影响，去年经济增长没有达到预期目标，固定资产投资增速放缓；不少工业企业生产经营遇到较大困难，工业经济运行质量效益不高，骨干龙头企业数量偏少；财政收入低速增长，收入结构不尽合理；城镇化水平仍然较低，城乡协调发展差距明显，中心城市辐射带动作用不强；环境保护任务艰巨，节能减排压力较大；改善民生与群众的期盼还有差距，影响安全稳定的因素仍然较多等等。对此，我们将采取措施努力加以改进。

　　二、2015年主要工作目标和任务

　　2015年是完成“十二五”规划的收官之年，也是贯彻十八届四中全会精神、全面推进依法治市的开局之年。当前，我市正处于保持适当速度、继续扩张总量的追赶期，促进转型跨越、不断深化改革的攻坚期，突破瓶颈制约、彰显相对优势的潜能释放期。虽然我国经济发展已进入以速度变化、结构优化、动力转换为主要特点的新常态，但经济发展总体向好、仍处于重要战略机遇期的基本面没有改变；虽然我市发展仍面临一系列的问题、困难和挑战，但也有着多项政策叠加、自然资源丰富、发展基础牢固、新增长点较多等鲜明优势；虽然近年来经济增长的速度放缓，但有利于我们以此为契机优化结构、升级产业、转变方式，提升发展的质量和水平。只要我们抢抓机遇，顺势而为，科学认识新常态，主动适应新常态，积极引领新常态，就一定能够在新一轮发展中赢得主动。

　　今年全市经济社会发展的主要预期目标是：生产总值增长11%左右；固定资产投资增长23%；地方财政收入同口径增长12%；城镇登记失业率控制在4%以内；城乡居民收入增长11%和13%；人口自然增长率控制在6.2％。以内；万元GDP能耗下降3.8%，市区二级以上优良天数达到190天以上。

　　需要说明的是，新常态下的发展目标，是一个预期性、区间性、引领性目标，体现了我们跳起来摘桃子，自我加压、加快发展的信心和决心。实现上述目标，一定要牢牢把握稳中求进的总基调，增强定力，保持耐心，主动作为，在有质量、有效益、可持续的前提下全力推动跨越发展；一定要把调结构、转方式作为一条主线，不断提升产业发展层次，打造渭南经济发展的升级版；一定要把深化改革、创新驱动作为根本动力，以改革开放、科技创新加快动力转换，形成稳健、可持续的增长格局；一定要把切实改善民生、增加群众福祉作为基本出发点，凝聚发展正能量，让人民群众真正成为渭南建设的参与者和共享者。

　　重点抓好以下几方面工作：

　　（一）坚持把狠抓项目、扩大投资作为重要支撑，持续增强发展动力

　　强化项目策划储备。紧盯中省政策取向和市场导向，紧扣“一带一路”建设和黄河金三角规划的实施，高质量策划储备一批重大产业、基础设施和民生类项目，确保年内策划重大前期项目200个以上。加强与中省部门的沟通衔接，争取更多项目挤进中省计划盘子和“十三五”规划，为投资持续增长打好基础。

　　扎实推进重大项目。坚持分级管理、分类推进的原则，完善项目推进机制，提高项目开工率和投产率，确保300个市级重点项目年度完成投资1464亿元。重点抓好西南铁路复线、黄韩侯铁路、沿黄公路等工程建设，确保年内韦罗高速、渭玉高速建成通车，蒲白高速完成路基工程，抽黄供水工程基本建成。力促黄河禹门口大桥、富平热电联产等项目上半年开工建设。

　　切实加大招商力度。围绕装备制造等支柱产业链延伸和3D打印、新能源新材料等新兴产业发展，紧盯央企、省企和东部产业转移，着力引进战略性投资者。强化领导干部带头招商，积极开展海外招商，着力提高招商引资质量。全面清理规范招商引资税收优惠政策，营造公平的市场环境。狠抓签约项目落实，提高项目开工率和资金到位率，确保招商引资到位资金突破600亿元。

　　持续优化投资环境。深化投融资体制改革，推广PPP投资模式，降低投资准入门槛，引导更多社会资本投向基础设施、环境保护、民生改善等领域。全面落实小微企业扶持政策，激发全民创业活力，推动民营企业和非公经济快速发展。进一步优化市民综合服务中心运行机制，全面推行服务承诺、限时办结等制度，持续开展服务企业单位评议工作，倒逼职能部门提高服务质量。加强社会诚信体系建设，严肃查处典型案件，确保投资发展环境根本改善。

　　（二）坚持把调结构、转方式作为重要任务，提升产业整体水平

　　推进工业结构优化升级。坚持工业强市不动摇，依靠存量稳增长，围绕增量调结构，尽快形成多点支撑的工业发展格局。一是全力支持优势骨干企业发展壮大。推动蒲城70万吨烯烃、富平生态水泥、青岛啤酒一期、韩城龙门煤化等项目尽快释放产能，加快澄城烟厂技改、舜天能源重组、金钼股份采选冶升级改造、大荔锂离子电池、卤阳湖卓达绿色建材等项目建设。认真研究解决煤炭行业当前遇到的困难，采取煤电联营、技术创新等措施，促进煤炭稳产促销。二是加快推动传统产业转型升级。落实企业“直供电”政策，制定企业技术改造、流动信贷、物流销售等补贴政策，积极推进煤化工、有色冶金及装备制造、光伏、食品加工等产业链工程，推动传统产业向中高端迈进。三是大力培育战略性新兴产业。加快3D打印产业培育基地建设，落实创投基金一揽子项目计划，着力引进发展3D打印与生物医疗器械产业化项目，加快3D打印在文化、旅游、教育等领域推广应用。积极推广电动新能源汽车，力求在引进电动汽车核心技术及配套生产项目上实现突破。有序推进智慧城市试点工作，加快以光网工程为重点的信息基础资源建设，逐步完善互联互通、资源共享的信息化服务体系。积极培育扶持大数据、物联网、云计算等示范效应明显的信息产业项目，以信息化带动工业化。四是着力提升工业园区发展水平。强化基础设施和功能配套，支持园内企业开展技术创新，真正将园区打造成工业发展的聚集区和示范区。全市新增规模以上工业企业50家，规模以上工业企业完成增加值680亿元，增长14%。

　　加快农业发展方式转变。坚持走产出高效、产品安全、资源节约、环境友好的现代农业发展道路，加快农业规模化、品牌化、标准化、市场化建设，夯实农业发展基础。认真落实耕地保护目标责任制，确保粮食总产稳定在200万吨以上。抓住省上实施“十百千万”工程的机遇，引导土地经营权有序流转，大力培育新型农业经营主体。持续推进现代农业园区、陕西现代畜牧业示范区和现代果业强市建设，力争新增市级以上农业园区10个，总面积达到75万亩。水果和设施蔬菜分别发展到305万亩和105万亩，畜牧业产值增长20%以上。依托滩涂资源，积极扶持发展水产养殖业，尽快形成农业发展新的增长点。大力发展具有地方特色的农副产品加工业和服务业，支持龙头企业发展壮大，鼓励发展休闲农业和乡村旅游，年内新增市级以上龙头企业20户。拓展提升“大荔模式”，完善现代农业服务体系。加强水利基础设施建设，着力解决中部易涝区排涝排碱问题，加快改善渭北旱塬灌溉条件。推进高标准基本农田建设，发展节水灌溉14万亩，改造基本农田17.1万亩。建立市县两级农产品质量安全追溯平台，加强农业园区农产品质量安全监测网络建设，实行农产品“三品一标”认证，提高农产品质量安全水平。

　　大力发展三产服务业。加大政策支持力度，完善考核奖励办法，着力提高三产服务业对经济的贡献率，确保三产增加值增长10%以上。一是持续推进大华山旅游目的地建设。坚持文化与旅游融合发展，策划启动20个文化旅游产业项目，确保沿黄公路洛河桥年底前建成通车。加快华山、少华山、洽川、仓颉庙、潼关古城以及“史记韩城·风追司马”等景区提升，整体谋划和建设黄洛渭三河文化和山水风貌大景区，推进唐桥陵、泰陵等保护开发，积极发展红色旅游，策划推出以华山为核心、辐射带动周边的精品旅游线路。强化市场营销，优化旅游环境，提升地接能力，推动市、县旅游联动发展。力争全年旅游综合收入达到280亿元，增长11%。二是加快商贸服务业提档升级。抓好经开区凯泰汽配五金机电城等项目建设，确保万达城市综合体8月开业运营、申华汽博园一期10月建成。编制电子商务发展规划，建立电子商务物流园区和孵化基地，培育30户年销售额过千万元的电子商务龙头企业，建设3-5个电子商务发展试点示范县。大力引进创新创业型服务组织，培育休闲、健康、养老、家政等新业态，促进消费持续增长。年内新增限额以上贸易企业80户，全社会消费品零售总额达到480亿元，增长14%。三是积极支持金融业发展。重视发挥银行融资主渠道作用，着力提高存贷比。推进投融资机制创新，设立政府引导基金，支持创投、风投基金参与产业结构调整和新兴产业发展。加强与资本市场对接，鼓励企业通过票据、债券、股权等多渠道融资，推动更多的企业上市。加快农商行组建，确保3家农信社改制进入实质性阶段。规范发展融资担保公司和小额贷款公司，重视发挥保险业作用，严厉打击非法融资，全面提升金融服务水平。

　　（三）坚持把统筹城乡、协调发展放在突出位置，着力推进新型城镇化

　　科学修编城镇规划。坚持城镇建设与自然山水相融合、城镇规划与人文历史相呼应，加快编制我市城市设计、风貌规划和新型城镇化发展规划。科学划定城镇边界，强化规划刚性约束，搞好富平经济社会发展规划、土地利用总体规划和城市规划“多规合一”试点。

　　突出中心城市发展。实施中心城市建设项目230个，年度完成投资225亿元。积极推动华县撤县设区，构建“一城四区”发展格局。优化城市路网，完善功能配套，着力解决制约城市发展的供水、供气、供热等问题，支持临渭区加快老城开发和城中村改造，增强中心区承载力和吸引力；以实施创新驱动战略为引领，以万达城市综合体开业运营为契机，推进新兴产业和现代服务业协调发展，进一步提升高新区发展层次；强化产业支撑、产城融合，加快光大农资和关中大红市场等建设，以商气聚人气，推动经开区突破发展。依托两条城中河，着力打造城市新亮点。其中渭河全面完成城区段右岸堤顶绿化、湭河入渭口景观绿化和滩区水生态景观核心区建设，加快南岸滨水东区建设；湭河以老街美食城二期建设为重点，加快两岸整体规划建设。加强城市精细化管理，启动全国文明城市创建，加大绿化、亮化和美化力度，不断提高中心城市首位度。

　　做优做美中小城镇。全面推广“人地房”相结合的征迁模式，推进城镇建设开发。以设施完善、功能配套、内涵提升、文明创建为重点，加快县城扩容提质。突出抓好重点镇建设，充分利用省上政策支持，加快5个省级重点示范镇和2个省级文化旅游名镇建设，确保年内完成投资10.3亿元。以新区建设、旧区整治和镇域综合改造提升为重点，扎实推进16个市级重点镇建设，努力打造县域副中心。

　　建好新型农村社区。坚持把新型农村社区建设作为推进城乡统筹的重要抓手，与移民搬迁、镇村综合改革和美丽乡村建设等有机结合，每个县市区年内启动建设5—10个新型农村社区，至少建成1个新型农村示范社区，让农民群众就近享受和城镇一样的生活。

　　推进农村转移人口市民化。放开城镇户口限制，推行人口居住证制度，将进城务工人员纳入城镇公共就业服务体系、住房和社会保障体系，做好养老、医保和子女教育等衔接工作，使进城落户人员融入城市、子女融入学校、家庭融入社区，确保农民进城落户10.8万人以上,全市城镇化水平达到42%以上。

　　（四）坚持把环境保护、生态文明作为最大的公共福利，加快建设美丽渭南

　　突出抓好环境保护。扎实推进“治污降霾·保卫蓝天”行动，建立治污降霾“三级”网格化管理机制，健全联防联控机制，年内渣土车全面安装GPS系统，中心城区机扫率基本实现全覆盖，县市区达到60%以上，努力让群众呼吸到更加清新的空气。认真做好主要污染物减排，加快实施重点行业和燃煤锅炉污染治理提标改造，确保14个中省减排项目按期建成、12家污水处理厂规范运行，煤炭消费量较上年削减3%。严格落实环保准入、资源环境总量控制等制度，坚守生态红线。扎实推进工业、建筑、交通和公共机构节能，关停小火电企业12家，淘汰水泥、电解铝等落后产能26万吨。坚持“铁腕促治”，实施新一轮渭河治理三年行动计划，确保地表水国控断面稳定达标。加强饮用水源地保护，建立水源污染事故防范预警应急体系，确保重点饮用水源地水质达标率100%。完善资源有偿使用和生态补偿制度，积极开展节能量和碳排放权、排污权、水权交易试点。

　　着力改善生态环境。统筹实施堤防、蓄滞洪区、湿地等生态工程建设，加快秦东大地园林化进程，搞好西潼、西禹、沿黄公路“大三角”绿色通道建设和新一轮退耕还林工作，强力整治取缔非法采石，推进矿山、采煤沉陷区和秦岭北麓生态恢复，完成植树造林35万亩，治理水土流失330平方公里。加快卤阳湖生态区建设，力争年内通过国家湿地公园验收。扎实推进渭河综合整治，全面完成堤防贯通、绿化及潼关入黄口景观建设等工程，确保渭河综合整治“五年计划”提前实现，让群众在生态持续改善中提高满意度、增强幸福感。

　　加快建设美丽乡村。按照“规划引导、环境整治、产业支撑、完善基础、健全制度”的思路，突出抓好108个示范村和339个重点村建设。扎实做好秸秆禁烧与综合利用工作，加快实施临渭、合阳、富平农村环境连片整治工程，确保年内通过省级审核。全面开展村庄人居环境整治，让群众在净化、绿化、美化家园中生活得更敞亮、更舒心。

　　（五）坚持把以人为本、改善民生作为基本落脚点，努力增进群众福祉

　　始终把民之所盼、民之所望作为施政所向，持续加大民生投入，优先安排民生项目，加快解决民生欠账，切实做到保基本、兜底线、促公平、可持续，让改革发展成果更多地惠及全市人民，让人民生活得更加殷实、更加幸福。

　　千方百计扩大就业。以高校毕业生、复员退伍军人、进城落户农村居民和就业困难人员为重点，认真落实扶持就业各项政策，适度扩大和提高小额担保贷款扶持范围和额度，加大技能培训力度，以创业促就业，开发公益性岗位，确保新增城镇就业5.2万人。

　　提升社会保障水平。认真做好农民工、灵活就业人员社会保障工作，健全实施好农民工工资保障金制度和城乡居民丧葬费补助制度，大力推进殡葬改革。强化社会救助体系，改进城乡低保和农村五保工作，完善农村留守儿童、妇女、老年人关爱政策，健全残疾人权益保障制度，支持社会福利和慈善事业加快发展。启动机关事业单位养老保险改革，全面推进社会化养老工作，启动建设市县残疾人康复中心、托养中心。

　　改善困难群众生活条件。统筹推进采煤沉陷区治理、棚户区改造和保障性安居工程建设，制定购买商品房补充保障房措施，新建各类保障房5.3万套。坚持精准扶贫，积极推广“参股建园”、“参股融资”等产业扶贫模式，高度重视解决返贫问题。持续推进扶贫移民搬迁七年行动计划，年内完成1.4万人、启动2.3万人的搬迁工程，实现脱贫18万人，解决20万人的安全饮水问题。

　　积极发展社会事业。加快实施第二期学前教育三年行动计划，新建改建公办幼儿园77所，其中中心城市新建10所。继续深化大学区改革，试点中小学校长职级制。加快实施“千校达标”工程，力促临渭、华阴、白水、潼关通过“双高双普”省级验收。推进中等职业教育资源整合，大力发展职业教育。深化公立医院改革和药品供应保障体系改革，探索建立分级诊疗和双向转诊制度，推进全科医生和居民契约服务模式。加快市图书馆建设，确保市博物馆6月底前对外开放，力争国家公共文化服务体系示范区创建年内通过验收。加强市体育中心和文化艺术中心管理运营，广泛开展各类活动，丰富群众文体生活。突出科学性、前瞻性，扎实做好“十三五”规划编制工作。

　　切实加强安全稳定。认真贯彻新《安全生产法》，推进政府监管网格化、企业管理精细化、行业直管专业化，构建无缝隙安全责任链条。始终以“零容忍”的态度抓好隐患排查整治，持续推进烟花爆竹、煤矿和非煤矿山整合关闭工作，强化日常监督检查，坚决杜绝重特大事故发生。加强社会治理制度创新，坚持以法治的思维处理信访问题，妥善化解各类矛盾纠纷，扎实推进平安渭南建设，健全社会治安防控体系，确保全市社会大局稳定。

　　持续推进双拥共建。一如既往地支持驻渭部队建设，深入开展国防教育，加快推进军民融合，健全国防动员体系，搞好预备役部队和民兵建设，争创国家双拥模范城“五连冠”，不断开创双拥共建工作新局面。

　　（六）坚持把深化改革、推进创新作为根本动力，激发发展生机活力

　　深入推进各项改革。继续推进简政放权，进一步深化行政审批制度改革，力争全市行政审批事项再减少10%左右，以权力“瘦身”为廉政“强身”。进一步支持韩城搞好计划单列试点，加快推进事业单位分类改革、政府向社会力量购买服务及县乡公务用车改革。推进镇村综合改革和农村土地确权登记工作，搞好第九次村民委员会换届选举工作。充分发挥市场在资源配置中的决定性作用，支持国有经济和民营经济融合发展，壮大混合所有制经济。继续深化市属国有企业改革，推行企业经营层年薪制。深化工商登记制度改革和财税改革。积极转变财政对经济发展的支持方式，更多地通过产权和股权投资、设立创投基金，吸引社会资本聚集，支持企业发展壮大。坚持节约集约利用土地，开展闲置土地清理工作，继续做好城乡建设用地增减挂钩和工矿废弃地复垦试点。

　　积极扩大开放合作。加快推动西渭融合发展，积极争取西安至渭南和华阴、大荔高铁公交化运营。加强对外交流合作，支持建设富平“中哈苹果友谊园”。积极参与丝绸之路经济带建设，全面实施黄河金三角区域合作规划，精心办好第三届黄河金三角投资合作交流大会和秦商大会，不断拓展发展空间。以渭南海关开关为契机，积极实施边疆、沿海港铁联运，构建东西货运大通道，全面落实支持进出口贸易企业相关政策，提高经济外向度。力争新增进出口备案企业30户以上，实现进出口总额2亿美元。

　　推进创新型渭南建设。积极实施科技统筹创新五大工程，加快以企业为主体的创新体系建设，建立市级以上科技研发平台10个，力争省级重大科技成果中试基地落户我市。鼓励民间资本参与科技服务和风险投资，探索科技贷款风险补偿和知识产权质押融资模式，积极破解科技投入瓶颈制约。深入推进校地校企合作，发挥好市科技资源统筹中心作用，力争技术合同交易额达到1.5亿元，专利授权量达到650件，全社会科技研发投入占GDP比重达到1.35%。创新方式、搭建平台，不断加强引才、育才和用才工作，加快实施三个“百人计划”，大力引进行业领军人才和部省高层次人才（团队），为产业转型升级提供强大智力支撑。

　　三、以依法行政为核心全面加强政府自身建设

　　认真贯彻落实市委《关于推进依法治市的若干意见》，坚持用法治的思维、法治的方式推进工作，加快建设职能科学、权责法定、执法严明、公开公正、廉洁高效、守法诚信的法治政府。

　　强化法制教育，推进全民守法。坚持领导干部带头学法、模范遵法，健全行政机关工作人员学法用法制度，崇尚法治信仰，增强法治意识, 养成依法办事的行为习惯。加大全民普法宣传力度，扎实推进“法律六进”活动，大力弘扬社会主义核心价值观，用法治文化教育社会民众，用法治思想引导社会舆论，提高全民对法律的认知度和遵从度，形成全社会遵法、守法、用法、护法的良好氛围。

　　依法严格履职，提高执法效能。健全公众参与、专家论证、风险评估、合法性审查等科学民主决策制度, 完善政府法律顾问制度，探索建立责任追究和倒查机制，坚决防止越权决策、随意决策和违法决策；坚持法定职责必须为、法无授权不可为，着力推进机构、职能、权限、程序、责任法定化，加快推行权力清单、责任清单和负面清单制度，坚决纠正不作为、乱作为和慢作为；深化行政体制改革，进一步理顺行政执法体制，探索跨部门、跨区域联合执法，坚决克服交叉执法、多头执法和低效执法；完善行政执法程序, 全面落实行政裁量权基准、重大执法审核、执法人员资格管理和持证上岗等制度，引入第三方机构对行政效能进行评估，建立领导干部干预行政执法记载制度，坚决杜绝以言代法、以权压法和徇私枉法，确保严格规范公正文明执法。

　　加强监督制约，规范权力运行。强化政府内部的制约与监督，对权力集中的部门和岗位，逐步实行分事行权、分岗设权、分级授权。对公共资金、国有资产、国有资源和领导干部履行经济责任情况，逐步实行全覆盖审计。加强行政监察，完善政府督查体系，建立行政复议纠错、行政诉讼败诉报告制度，落实行政问责制。依法接受市人大及其常委会的监督，主动接受市政协的民主监督，广泛听取民主党派、工商联、无党派人士、各人民团体的意见，重视司法、舆论和社会监督，全面推进政务公开，让权力在阳光下运行。

　　坚持廉洁从政，持续改进作风。认真践行“三严三实”，讲政治、守规矩，时刻做到心中有党、心中有民、心中有责、心中有戒。巩固拓展党的群众路线教育实践活动成果, 狠抓整改落实，健全制度机制，推进作风建设常态化、长效化。坚持从严治权、从严治官、从严治“一把手”，对懒政庸政怠政、不作为的严肃问责，对不敢抓不敢管、混日子的干部，坚决采取组织措施。严格执行新《预算法》，大力倡俭治奢，政府所有收支一律纳入预算管理，所有公共资金一律接受审计监督，确保财政供养人员只减不增，会议、文件持续减少，“三公”经费支出明显下降。扎实推进政府系统党风廉政建设,加强绩效考核和效能监察，严肃查处各类违法违纪案件，以实际行动赢得人民群众的拥护和信任。

　　各位代表，我们深知，人民群众对渭南发展充满着期待和向往；我们也深知，千里之远需跬步以至，发展大业在日积月累。建设富裕文明和谐活力的陕西东大门，需要我们从改革发展的一点一滴做起，从群众期盼的一情一事做起，从每个人的一言一行做起。让我们在市委的坚强领导下，低调务实、埋头苦干、砥砺奋进、合力向前，共同创造渭南更加美好的明天!
